# Localization of neurones expressing the gap junction protein Connexin45 within the adult spinal dorsal horn: a study using Cx45-eGFP reporter mice

**DOI:** 10.1007/s00429-012-0426-1

**Published:** 2012-05-26

**Authors:** R. J. Chapman, V. K. Lall, S. Maxeiner, K. Willecke, J. Deuchars, A. E. King

**Affiliations:** 1 Institute for Membrane and Systems Biology, University of Leeds, Leeds, LS2 9JT UK; 2 Institute of Genetics, University of Bonn, 53117 Bonn, Germany

**Keywords:** Immunohistochemistry, Dorsal horn, Connexin 45, Gap junction, Nociception

## Abstract

Connexin (Cx) proteins localized to neuronal and glial syncytia provide the ultrastructural components for intercellular communication via gap junctions. In this study, a Cx45 reporter mouse model in which the Cx45 coding sequence is substituted for enhanced green fluorescent protein (eGFP) was used to characterize Cx45 expressing neurones within adult mouse spinal cord. eGFP-immunoreactive (eGFP-IR) cells were localized at all rostro-caudal levels to laminae I–III of the dorsal horn (DH), areas associated with nociception. The neuronal rather than glial phenotype of these cells in DH was confirmed by co-localisation of eGFP-IR with the neuronal marker NeuN. Further immunohistochemical studies revealed that eGFP-IR interneurones co-express the calcium-binding protein calbindin, and to a lesser extent calretinin. In contrast, eGFP-IR profiles did not co-localize with either parvalbumin or GAD-67, both of which are linked to inhibitory interneurones. Staining with the primary afferent markers isolectin-B4 (IB4) and calcitonin gene-related peptide revealed that eGFP-IR somata within laminae I–III receive close appositions from the former, presumed non-peptidergic nociceptive afferents of peripheral origin. The presence of 5-HT terminals in close apposition to eGFP-IR interneuronal somata suggests modulation via descending pathways. These data demonstrate a highly localized expression of Cx45 in a population of interneurones within the mouse superficial dorsal horn. The implications of these data in the context of the putative role of Cx45 and gap junctions in spinal somatosensory processing and pain are discussed.

## Introduction

In the adult mammalian central nervous system (CNS) gap junctions provide for rapid metabolic and electrical inter-cellular communication between neurones and glia (Nagy and Rash 2000). Gap junctions are composed of two hemi-channels expressing homotypic, heterotypic or heteromeric combinations of specific connexin (Cx) subunit proteins that influence channel conductance and kinetics (Sohl and Willecke 2003). Profiling studies in brain indicate that Cx subtypes are differentially localized to glia and neurones thus Cx36 and Cx45 are unambiguously present in neurones (Condorelli et al. 1998; Maxeiner et al. 2003; Hombach et al. 2004) whereas Cx43 and Cx30 are widely present in astrocytes (Theis et al. 2005). The concept of functional compartmentalization has emerged whereby the expression pattern of Cx proteins defines the strength of coupling between different cell types, thereby establishing a neuron-glial syncytium that supports behaviours such as synchronized firing or propagation of calcium waves across disparate neuronal populations (Dermietzel 1998). Understanding the identity and expression patterns of Cx proteins is therefore a prerequisite to study the functional significance of gap junctions in the mature CNS.

Few studies have described in detail the distribution of gap junctions in adult rat spinal cord, but freeze-fracture studies revealed an abundance of mixed electrical/chemical synapses distributed across the grey matter of laminae III–IX (Rash et al. 1996). Regarding specific Cx subtypes, spinal cord expression profiles to date parallel those reported for brain. Thus, neuronal gap junctions contain Cx36 (Rash et al. 2000) or Cx47 (Teubner et al. 2001), whilst Cx32 and Cx43 are associated principally with oligodendrocytes and astrocytes, respectively (Rash et al. 2000; Nagy and Rash 2000; Ochalski et al. 1997). More recently, the use of *Lac*Z reporter gene expression or targeted Cx45 gene deletion in mice has revealed a strong association of this Cx subtype with neurones that express the protein marker NeuN (Sohl et al. 2005). Cx45 is highly expressed in brain during embryogenesis and after birth, thereafter levels decline such that Cx45 is more restricted, e.g. to thalamus or hippocampal CA3 region and different cortical layers (Maxeiner et al. 2003). To our knowledge, a full characterisation of the distribution of any of the neuronal Cx subtypes, including Cx45, throughout the murine spinal cord has not been performed. Determining the identity and distribution of the connexins in the dorsal horn is of interest because recent data imply a role for gap junctions localized to neurones or glia in rhythmic activity manifest in rat dorsal horn circuitry in vitro (Asghar et al. 2005; Chapman et al. 2009) and gap junctions are implicated in pain facilitation and inflammation-induced nociceptive behaviours (Lan et al. 2007; Qin et al. 2006).

Determining the distribution of Cx proteins in the CNS using traditional approaches of in situ hybridisation and immunohistochemistry has provided variable results and connexin-specific protocols required for both methods are not readily amenable to dual labelling (Sohl et al. 2005). As an alternative strategy, we have used a previously characterized transgenic mouse mutant line that expresses green fluorescent protein (GFP) in place of the *Cx45* coding sequence (Maxeiner et al. 2005) to reveal a discretely localized population of Cx45 expressing cells within the superficial layers of the spinal dorsal horn. Immunohistochemistry was used to define the phenotype of neurones expressing Cx45 and determine putative sources of extrinsic innervation. The concentrated expression of these cells within laminae I–III places them in a strategic position to potentially influence the processing of somatosensory afferent inputs, particularly those arising from nociceptors.

## Materials and methods

### Animals

All procedures were carried out in accordance with the UK Animals (Scientific Procedures) Act of 1986 and experimental protocols were approved by the local Faculty of Biological Sciences Ethics Committee. All experiments utilized mice with a cell-directed deletion of Cx45 and a concomitant activation of GFP (Theis et al. 2000), the constructs of which have been outlined previously in detail (Maxeiner et al. 2005). In summary, to allow for conditional deletion of the Cx45 coding region on exon3 of the Cx45 gene, exon3 was flanked by two loxP sites in intron2 and downstream of exon3, respectively. Upstream of the 5′ prime loxP site, a frt sites flanked minigene harbouring the neomycin resistance gene under the regulatory elements of the phosphoglycerate kinase promoter (PGK-neo) has been inserted to identify positive clones during ES-cell culture. Downstream of the second loxP site, the immediate upstream intronic sequence of the Cx45 gene has been duplicated and the endogenous Kozak consensus motif has been fused to the enhanced green fluorescent protein (eGFP) coding DNA. Eventually, Cre-recombinase expression under control of the nestin promoter will lead to the recombination of both loxP sites and delete the Cx45 coding DNA by replacing it with eGFP, restricting eGFP expression primarily to neurones as described previously (Maxeiner et al. 2005). To avoid putative disturbances in the expression of Cx45/eGFP by constitutive expression of the neomycin resistance gene, all mice analysed were devoid of PGK-neo minigene accomplished by previous breedings to Flp-recombinase expressing mice.

### Cx45-eGFP immunohistochemistry

Adult mice (*n* = 6) were anaesthetised with pentobarbitone (60 mg/kg, i.p.) and transcardially perfused with fixative containing 4 % paraformaldehyde (PFA; in 0.1 M phosphate buffer, pH 7.4). The spinal cords were removed and post-fixed in 4 % PFA overnight at 4 °C. Transverse sections (50 μm) cut from the middle of the cervical, thoracic, lumbar and sacral regions were cut on a vibrating microtome (Leica, Milton Keynes, UK) and collected into phosphate-buffered saline (PBS; pH 7.2). Sections were permeabilised by the inclusion of 0.1 % Triton X-100 (Sigma, UK) in the primary antibody solution to GFP, washed in PBS (3 × 10 min) and transferred to either chicken or rabbit anti-GFP primary antibodies (1:1,000, Invitrogen) for 12–36 h (4 °C). Prior to secondary antibody application, all sections were washed in PBS (3 × 10 min). Anti-GFP was visualised using Alexa^488^-conjugated donkey anti-chicken or rabbit (1:1,000, Invitrogen). Secondary antibodies were applied for 2–3 h (4 °C) and sections washed in PBS before drying, mounting and cover slipping using Vectashield (Vector Laboratories, Peterborough, UK). As a procedural control, the primary antibodies were replaced by PBS to ensure that the antiserum detected the appropriate antigen and under these conditions no staining occurred. Low- and high-power images were captured using a Nikon (Surrey, UK) Eclipse E600 epifluorescence microscope and AcQuis image capture software (Synoptics, Cambridge, UK). Corel Draw 13 was used to adjust brightness, contrast, intensity and gamma, if appropriate. Some images (Fig. 3) were inverted to aid visualisation of co-localisation in black and white images.

### Double immunolabelling protocol

Following incubation in anti-GFP antibodies and Alexa^488^-conjugated secondary antibodies as described above, spinal cord lumbar sections were washed in PBS (3 × 10 min) and incubated in primary antibodies (in PBS/0.1 % Triton X-100) for 12–36 h (4 °C). Sections were washed in PBS (3 × 10 min) prior to secondary antibody application. Anti-GFP was visualised using Alexa^488^-conjugated donkey anti-chicken or rabbit (1:1,000, Invitrogen) and all other antigens were individually visualised using Alexa^555^-conjugated to the appropriate species for 2–3 h (4 °C). Biotinylated IB4 (1:100) was visualised by incubating sections for 1 h in Streptavidin Alexa^555^ (1:100, Invitrogen). Staining for glutamate decarboxylase (GAD)-67 involved section pre-treatment by incubating in 50 % ethanol (30 min) and then in 10 % donkey serum (30 min), but with the avoidance of triton following the protocol of Fong et al. (2005) who noted that detergent decreased GAD-67 labelling in somata in the brainstem. Thereafter, sections were incubated in primary antibody for 12–36 h (4 °C). GAD-67 was visualised using a two-stage process: (1) sections were incubated in biotin anti-mouse (1:200, Vector Laboratories) for 4 h (4 °C), washed (3 × 10 min) and incubated in ExtrAvidin peroxidase (EAP; 1:1,500, Sigma) for 12 h (4 °C); (2) following removal from EAP, sections were washed (3 × 10 min each) and amplified using a tyramide signal amplification (TSA) kit (Coumarin–tyramide solution (1:100 PBS) plus 1 % hydrogen peroxide solution (Perkin Elmer LAS Inc., USA). Finally, sections were washed in PBS (3 × 10 min), air-dried, mounted and cover slipped using Vectashield for viewing with a Nikon E600 image capture system equipped with epifluorescence.

### Immunohistochemistry for Cx45

2-month-old wild type mice (C57/BL6) were anaesthetised with intraperitoneal pentobarbital solution (50 mg/Kg) and transcardially perfused with 0.1M PBS, pH 7.4 followed with 2 % PFA and 0.2 % picric acid in 0.1 M phosphate buffer (PB), pH 7.4 for 15 min. Spinal cords were sectioned at 30–50 μm as above. Sections were treated with pepsin (0.2 mg/ml) in 2 M HCL at 37 °C for 10 min followed by 3-, 10-min washes in PB (Vervaeke et al. 2010). Sections were then permeabilised with the inclusion of 3 % Triton X-100 (Sigma) in the primary antibody solution; polyclonal rabbit anti-Cx45 (1:250, Invitrogen, cat 40-7000) for 36 h at 4 °C before visualisation with Alexa^555^ conjugated donkey anti-rabbit (1:1,000, Invitrogen) as above. As a procedural control, the primary antibodies were replaced by PBS to ensure that the antiserum detected the appropriate antigen and under these conditions no staining occurred.

### Antibody characterisation

#### Calbindin (CB)

Mouse anti-CB D-28k monoclonal antibody (Swant, Switzerland; code no. 300, lot no. 07(F); form, lyophilised concentrated supernatant with no preservatives, used at 1:5,000) was produced by hybridization of mouse myeloma cells with spleen cells from mice immunized with CB purified from chicken gut. The staining observed with this antibody gave an identical staining pattern in the mouse spinal cord compared with previous investigations using an antibody from a different origin (Ruda et al. 1994) and did not stain the brain of CB knockout mice (Swant Data sheet).

#### Calcitonin gene-related peptide (CGRP)

Chicken anti-CGRP antibody was raised against the synthetic peptide sequence KDNFVPTNVGSEAF-NH2 (Neuromics, code no. CH14100, used at 1:200). Staining of spinal cord sections revealed patterns previously documented with this antibody (Gao et al. 2009) and is identical to immunoreactivity observed with different antibodies to CGRP (Neumann et al. 2008).

#### Choline acetyltransferase (ChAT)

Polyclonal goat anti-ChAT antibody was produced from human placental enzyme (Millipore, code no. AB144P, used at 1:500). Immunoblot analysis has identified antibody specificity in cholinergic neurones in the CNS in a variety of species (Millipore data sheet) and stains brainstem and spinal cholinergic neurones in well established patterns (Milligan et al. 2006).

#### Calretinin (CR)

Rabbit anti-CR polyclonal antibody (Swant, Switzerland; code no. 7699/3H, lot no. 18299; form, lyophilised antiserum, used at 1:5,000) was produced against human recombinant CR. Antibody specificity was evaluated by Biotin-Avidin labelling of fixed brain preparations and by immunoenzymatic labelling of immunoblots (Swant data sheet; Schwaller et al. 1993) and does not stain the brain of CR knockout mice (Swant data sheet).

#### Cx45

**Fig. 1:**
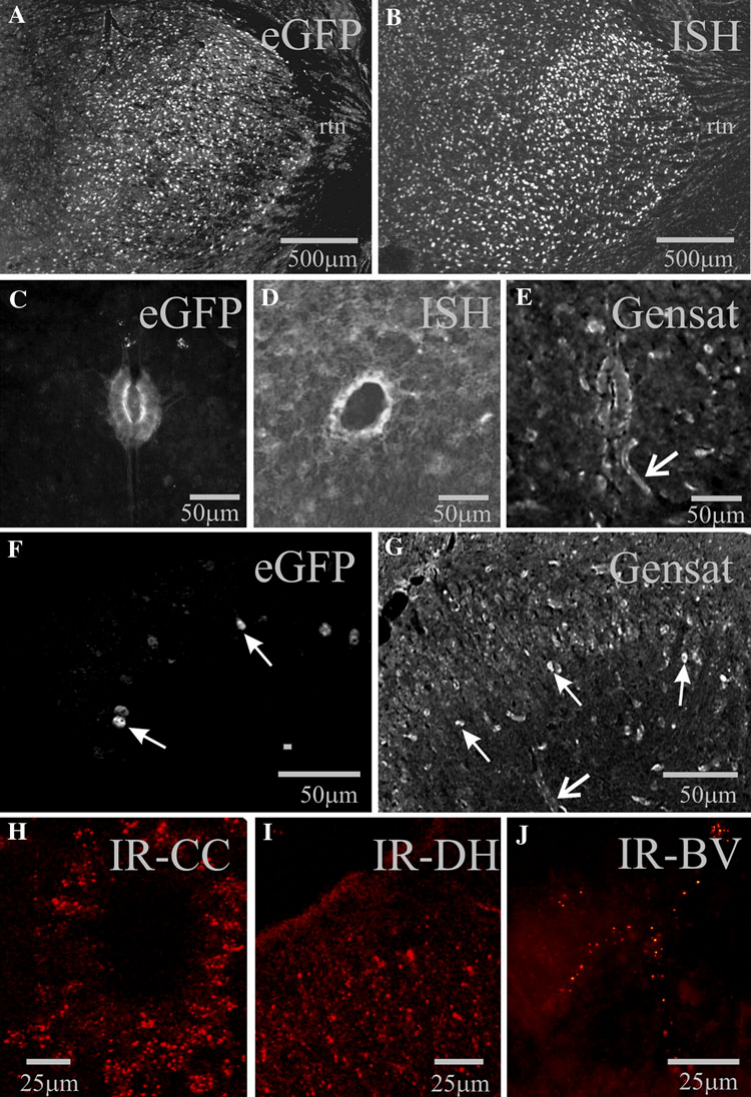
Regional expression of Cx45-eGFP is consistent with in situ hybridisation, reporter expression in another transgenic mouse line and protein localisation. **a**, **c**, **f** eGFP-IR from the Cx45-eGFP mouse. **b**, **d** In situ hybridisation images for Cx45, taken from the Allen Brain Atlas. **e**, **g** Images of expression in a Cx45 BAC reporter mouse taken from the Gensat project. High levels of expression are evident in neurones in the thalamus (**a**, **b**), ependymal cells surrounding the spinal cord central canal (**c**–**e**) and in layers I–III in the dorsal horn (**f**, **g**). *Closed head arrows* indicate neurones whilst *open head arrows* blood vessels apparent due to smooth muscle labelling. Immunoreactivity for Cx45 can also be detected in the ependymal cell layer (**h**), dorsal horn (**i**) and in blood vessels (**j**). **b**, **e**, **g** Inverted from original images to correspond with those shown here.* rtn* reticular thalamic nucleus. Colour images are available online

Rabbit anti-Cx45 is raised against a synthetic peptide corresponding to the c-terminal region of protein (Invitrogen code no. 40-7000, used at 1:250). The manufacturer indicates that it specifically detects Cx45 in mouse brain homogenates in western blots and stains expected cell types in retina and mouse brain. Staining was comparable to expression as indicated in Fig. 1 in this study.

#### GAD-67

Mouse anti-GAD67 monoclonal antibody (Chemicon, MAB5406; Clone 1G10.2, used at 1:1,000) was produced against recombinant GAD-67 fusion protein. The GAD-67 antibody reacts with the 67 kDa isoform of GAD-67 of rat, mouse, and human origins, and there is no detectable cross reactivity with GAD-65 by western blotting of rat brain lysate (Chemicon data sheet for MAB5406; Fong et al. 2005).

#### Glial fibrillary acidic protein (GFAP)

Mouse monoclonal anti-GFAP antibody was produced by hybridisation of Sp2/0 myeloma cells with splenocytes from Balb/c mice immunised with purified human GFAP (Biomol, code no. BML-GA1170; clone EB4). The antibody recognises GFAP in a wide range of species (Biomol data sheet) and staining displays classic morphology and distribution of fibrillary astrocytes.

#### GFP

According to the manufacturer, both rabbit (Invitrogen, A11122) and chicken (Invitrogen, A10262) anti-GFP antibodies are suited for detection of native GFP, GFP variants, and GFP fusion proteins by western blot and immunohistochemistry analysis using transgenic mice expressing the GFP gene product. Labelling was only detected in cells that expressed GFP and no staining was observed in sections derived from wild-type mice.

#### 5-Hydroxytryptamine (5-HT)

Rabbit polyclonal anti-5-HT antibody was raised against 5-HT coupled to bovine serum albumin with PFA (used at dilution of 1:1,000). The pattern of staining observed matches that observed with other 5-HT antibodies in spinal cord terminals and brainstem raphe neurones (Izzo et al. 1993).

#### IB4

Isolectin GS-IB4 from Griffonia simplicifolia conjugated to Alexa^568^ (Invitrogen, I21412) was used (1:100) to label non-peptidergic nociceptive afferents (Snider and McMahon 1998).

#### Mu opioid receptor (MOR)

Monoclonal rabbit anti-MOR antibody was raised against a synthetic peptide corresponding to residues on the human MOR (Abcam, code no. AB51140, diluted to 1,000). The MOR antibody recognised a single band of approximately 52 kDa on immunoblots from human brain lysate (Abcam data sheet). Staining was comparable to that observed using different antibodies to the receptor (Arvidsson et al. 1995).

#### Neuronal nuclei (NeuN)

Monoclonal mouse anti-NeuN (used at 1:1,000) was raised against purified cell nuclei from mouse brain (Chemicon, code no. MAB377; Clone A60). Immunohistochemistry and immunoblots in a variety of CNS tissues revealed detectable antigen only in nerve cells (Mullen et al. 1992). The immunohistochemical staining is primarily localized to the nucleus of the neurones, with lighter staining in proliferative zones (Mullen et al. 1992). The few cell types not reactive with MAB377 include Purkinje, mitral and photoreceptor cells.

#### Neurokinin-1-receptor (NK_1_R)

Polyclonal guinea pig anti-NK_1_R antibody was raised against a synthetic peptide corresponding to the C-terminal sequence (KTMTESSSFYSNMLA; amino acid residues 393–407) of the NK_1_R (Biomol, code no. BML-NA4200, used at 1:500). This antibody has been characterised extensively in rat (Vigna et al. 1994).

#### Nitric oxide synthase (nNOS)

Polyclonal rabbit anti-nNOS was raised against a synthetic peptide corresponding to aa1414–1434 of human brain NOS (NOS1; Biomol, code no. BML-SA227-0025, used at 1:1,000). This antibody specifically recognizes human, mouse and rat nNOS (Biomol data sheet). Antibody staining in mouse spinal cord reveals identical patterns to those previously documented (Maihofner et al. 2000; Ma and Eisenach 2007).

#### Oxytocin (OT)

Polyclonal rabbit anti-OT was raised against the amino acid sequence CYIQNCPLG, corresponding to N-terminal amino acids 20–28 of OT (Abcam, code no. AB11143, used at 1:10,000). According to the manufacturer this antibody blocks completely with OT peptide. Staining with this antibody revealed a similar pattern of distribution to previous investigations in the mouse spinal cord and a lack of staining was observed in knockout mice lacking OT (Robinson et al. 2002).

#### Protein kinase C (PKC)γ

Polyclonal rabbit anti-PKCγ was raised against a peptide mapping the c-terminus of PKCγ of mouse origin (Santa Cruz Biotech, sc-211, used at 1:1,000). Staining with this antibody gave an identical pattern of distribution in the mouse spinal cord to previous reports (Malmberg et al. 1997).

#### Parvalbumin (PV)

Monoclonal mouse anti-PV was produced by hybridization of mouse myeloma cells with spleen cells taken from mice immunized with PV purified from carp muscles (Swant, Switzerland; code no. 235, lot no. 10-11(F); form, lyophilised ascites, used at 1:5,000). Antibody specificity was evaluated by immunoenzymatic labelling of immunoblots and radioimmunoassay (Swant data sheet); (Celio et al. 1988). Additionally, immunolabelling using this antibody is absent in PV knockout mice (Schwaller et al. 1999).

#### Tyrosine hydroxylase (TH)

Polyclonal sheep anti-TH was raised against native rat TH purified from pheochromocytoma (Novus Biologicals, code no. NB300-110; Clone LNC1, used at 1:1,000). Staining with this antibody was identical to that observed in mouse spinal cord using an antibody from a different origin (Brumovsky et al. 2006).

### Confocal microscopy and 3D reconstruction

Mounted sections were viewed on a confocal laser scanning fluorescence microscope equipped with both argon (λ_ex_ = 488 nm) and He-Ne (λ_ex_ = 543 nm) lasers that are able to visualize optical sections through a 40× Fluor oil immersion objective (numerical aperture 1.3). Confocal images were captured as single slices or stacks and images analysed using Reconstruct™ software (Fiala 2005). Serial images were imported into Reconstruct™ and calibrated using a scale bar. A 3D model of Cx45-eGFP cell bodies could be generated by outlining the area of the cell somata within each serial section image. Only close appositions to Cx45-eGFP cell bodies were quantified since there is only sparse dendritic staining for eGFP. To be considered as a close apposition, the labelled presynaptic fibre was required to be directly adjacent to the GFP neurone in the same plane of focus, determined through confocal microscopy and rotation of Z-stack images of apparent contact point.

### Data and statistical analyses

Cell counts were calculated from the whole dorsal horn region of 10 representative sections from a minimum of 3 animals and contact density of potential terminal appositions were calculated from a minimum of 15 whole cells from each animal. Data are expressed as mean ± SEM where *N* represents the total number of cells and* n* represents the number of animals.

## Results

### Regional Cx45-eGFP expression in the CNS correlates to in situ hybridisation

To determine if regional expression of eGFP in the Cx45 mice was consistent with Cx45 gene expression, we compared eGFP immunohistochemistry with expression reported by the Allen Brain Atlas (http://www.brain-map.org/) in the brain (Image Series ID:77887876) as well as the spinal cord (Image series ID = 100009459). The expression studies reveal highest levels in the brain in the thalamic relay nuclei, whilst the reticular thalamic nucleus is devoid of labelling (Fig. 1b). This pattern is repeated in Cx45-eGFP mice, where eGFP immunoreactivity is strong in thalamic relay neurones but absent from the reticular nucleus (Fig. 1a). In the spinal cord Cx45-eGFP was apparent in the ependymal cells lining the central canal (Fig. 1c) and this is paralleled by the expression levels detected Allen Brain Atlas spinal cord (Fig. 1d). We further compared expression in this transgenic mouse to that observed in a reporter mouse made by the Gensat project (Gensat 2011) using a BAC vector to control expression of Cx45 (http://www.gensat.org/imagenavigator.jsp?imageID=80609). Expression was comparable, localised predominantly to the ependymal layer of the central canal (Fig. 1e) and the dorsal horn (Fig. 1f, g). In addition, the Gensat mouse revealed the expected labelling of blood vessel smooth muscle (Fig. 1e, g). This is not observed in the eGFP mouse as the deletion of Cx45 and hence expression of GFP is directed only to neurones since eGFP-positive cells co-labelled with NeuN but not GFAP (see below). We also compared expression with labelling for Cx45 protein and found concentrated immunoreactive spots, presumably gap junction proteins, in the ependymal cell layer (Fig. 1h), dorsal horn (Fig. 1i) and in blood vessels (Fig. 1j). Thus, the reporter expression pattern in neurones in the mouse used in this study was similar to that of another transgenic mouse, to expression revealed by in situ hybridisation and to the localisation of immunoreactivity.

### Cx45-eGFP cells within the spinal cord dorsal horn are neuronal

**Fig. 2:**
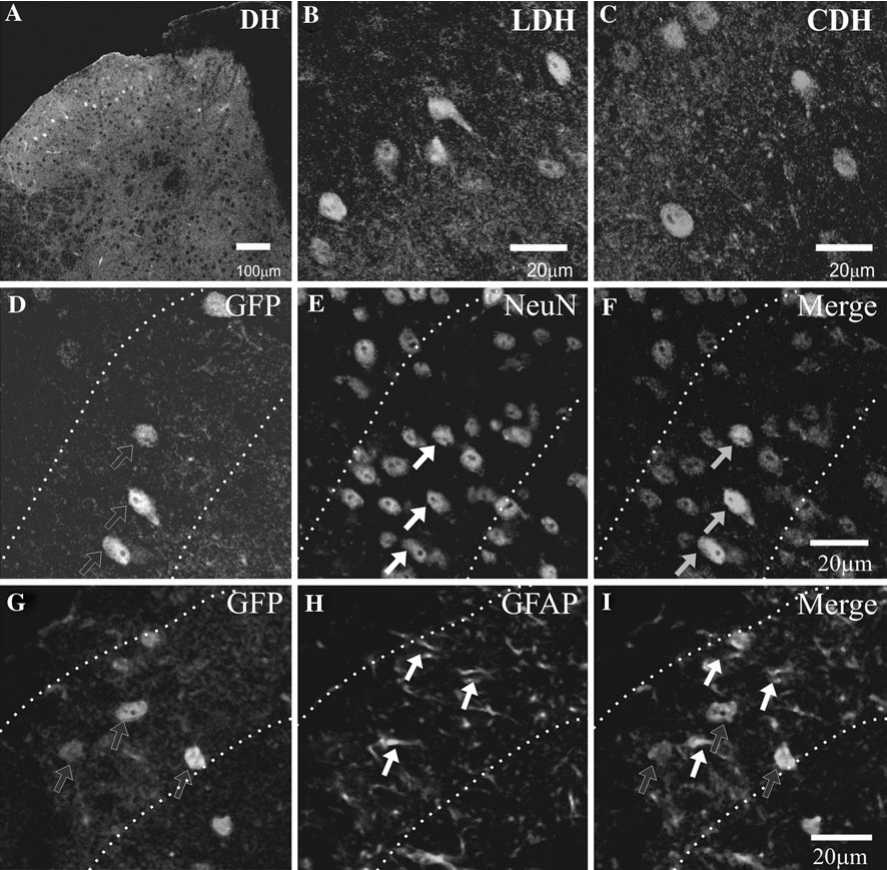
Distribution of Cx45-eGFP cells is mainly concentrated within neurones localized to laminae I–III of the cervical, thoracic, lumbar and sacral DH. **a** Confocal scan of the lumbar spinal DH showing a discrete band of GFP-IR cells lying predominantly within laminae II–III. High power magnification of Cx45-eGFP cells present in the lumbar DH (LDH; **b**) and cervical DH (CDH; **c**). **d**–**i** Confocal tiled scan shows Cx45-eGFP cells co-localize with the neuronal marker NeuN (**f**) but not with the glial marker GFAP (**i**) within laminae I–III. Cx45-eGFP cells, *black arrows with white borders*; NeuN and GFAP, *white arrows*. The *third tile of each row* shows a merged representation; GFP-IR in cells was co-localized with NeuN, *grey arrows*. *Dashed lines* represent the borders of lamina II

In addition to the ependymal cell layer, GFP-immunohistochemistry to reveal the distribution of Cx45-eGFP in the spinal cord indicated a discrete localisation to laminae I–III of the dorsal horn (Fig. 2a). The highest concentration of Cx45-eGFP cells was in lamina II encompassing both the inner and outer layers. Cx45-eGFP cells were also observed in lamina III, with most cells located near the laminae II–III border. Fewer Cx45-eGFP cells were found scattered across lamina I. No Cx45-eGFP cells were observed in deeper laminae of the dorsal horn or within the ventral horn. Abundant Cx45-eGFP-immunoreactive (eGFP-IR) could be found within the lumbar (Fig. 2b) and thoracic regions of the spinal cord, although numerous Cx45-eGFP cells were observed at all rostro-caudal levels including cervical (Fig. 2c) and sacral regions. 3D reconstruction of 90 Cx45-eGFP cells revealed a mean somatic surface area of 45.9 ± 7.5 μm^2^ (*n* = 6). Dual-immunolabelling of Cx45-eGFP with either NeuN or GFAP revealed a strong association with the former. Whilst the number of NeuN-IR cells was significantly higher than that of Cx45-eGFP cells, all eGFP-IR was co-localized with NeuN within laminae I–III (*n* = 3; Fig. 2d–f). In contrast to this, whilst GFAP itself was abundantly expressed within spinal grey matter, there was no apparent co-localisation with Cx45-eGFP-IR profiles (*n* = 3; Fig. 2g–i).

### Cx45-eGFP cells contain calcium-binding proteins

**Fig. 3:**
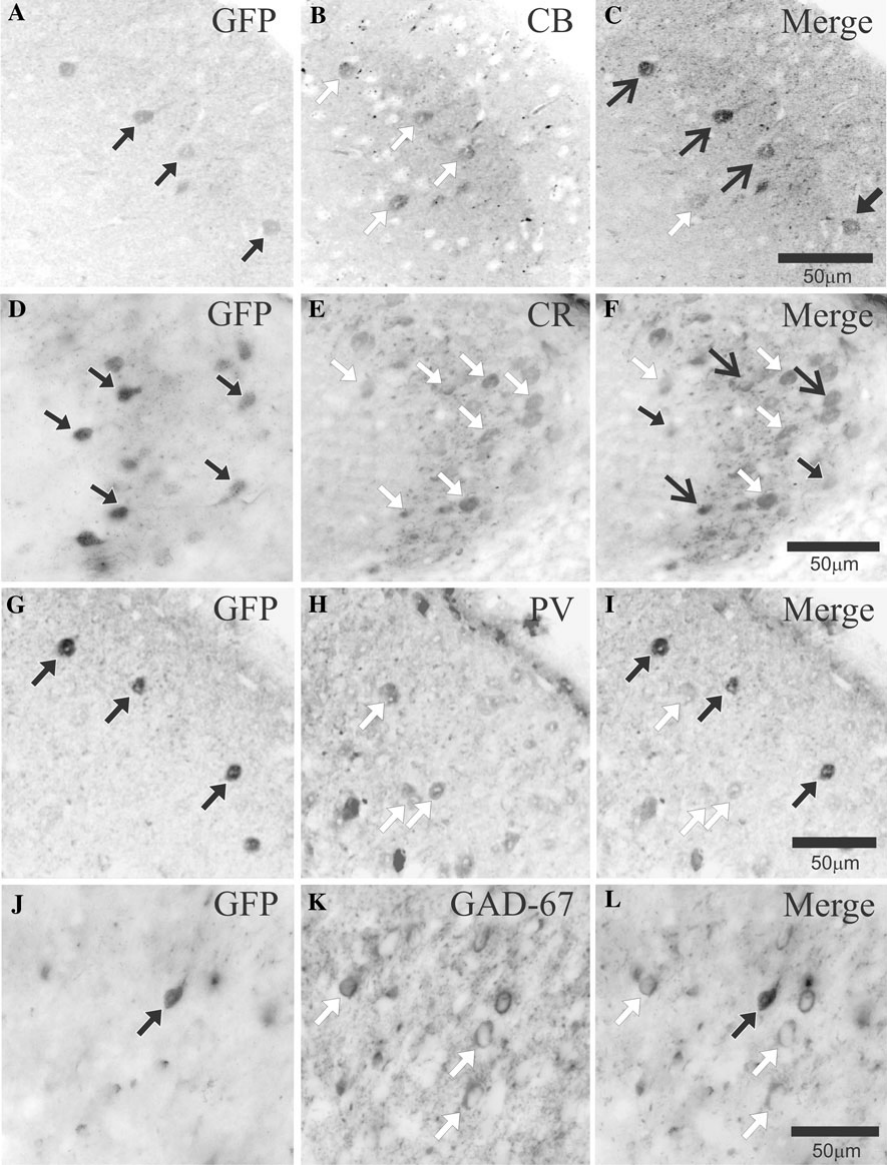
Cx45-eGFP cells co-localize with the CBPs CB and CR. **a**–**f** Cx45-eGFP cells show co-localisation with CB (**c**) and CR (**f**). **g**–**l** No co-localisation is observed with PV, or with GAD-67. Cx45-eGFP cells, *closed head black arrows*; cells containing CB, CR, PV or GAD-67, *white arrows*; cells displaying co-localisation, *open head black arrows*

To probe further the phenotype of the Cx45-eGFP cells, we performed dual immunolabelling of GFP with the calcium-binding proteins (CBPs), CB, CR and PV as well as the GABAergic marker GAD-67. All of the antigens investigated revealed a wide distribution of cells containing CB, CR, PV and GAD-67 throughout the dorsal horn and in significantly higher numbers than Cx45-eGFP cells (Fig. 3), in accordance with those published in the atlas of the mouse spinal cord (Watson et al. 2009). Immunoreactivity for the CBPs could be found throughout the spinal cord, with a higher percentage of ventral horn neurones staining for PV than CB or CR. In the dorsal horn, pronounced CB and CR immunoreactivity was observed within laminae I–II (Fig. 3b, e), whilst PV (Fig. 3h) intensely stains the inner part of lamina II. Of the Cx45-eGFP cells observed within laminae I–III (Fig. 3a, d, g, j), 93.5 ± 0.6 % were immunoreactive for CB (mean number of cells taken from 10 sections: 17 ± 1 GFP cells, and 16 ± 1 CB containing cells; *N* = 405 of 433, *n* = 3; Fig. 3c) and, to a lesser extent, 26.76 ± 1.2 % were immunoreactive for CR (mean number of cells from 10 sections: 19 ± 1 GFP cells, and 7 ± 1 CR containing cells; *N* = 110 of 411, *n* = 3; Fig. 3f). PV or GAD-67 containing cells were distinct from Cx45-eGFP cells at all levels of the dorsal horn (*n* = 3, Fig. 3i, l).

### Cx45-eGFP cells are putatively innervated by non-peptidergic sensory afferents and descending monoaminergic fibres

**Fig. 4:**
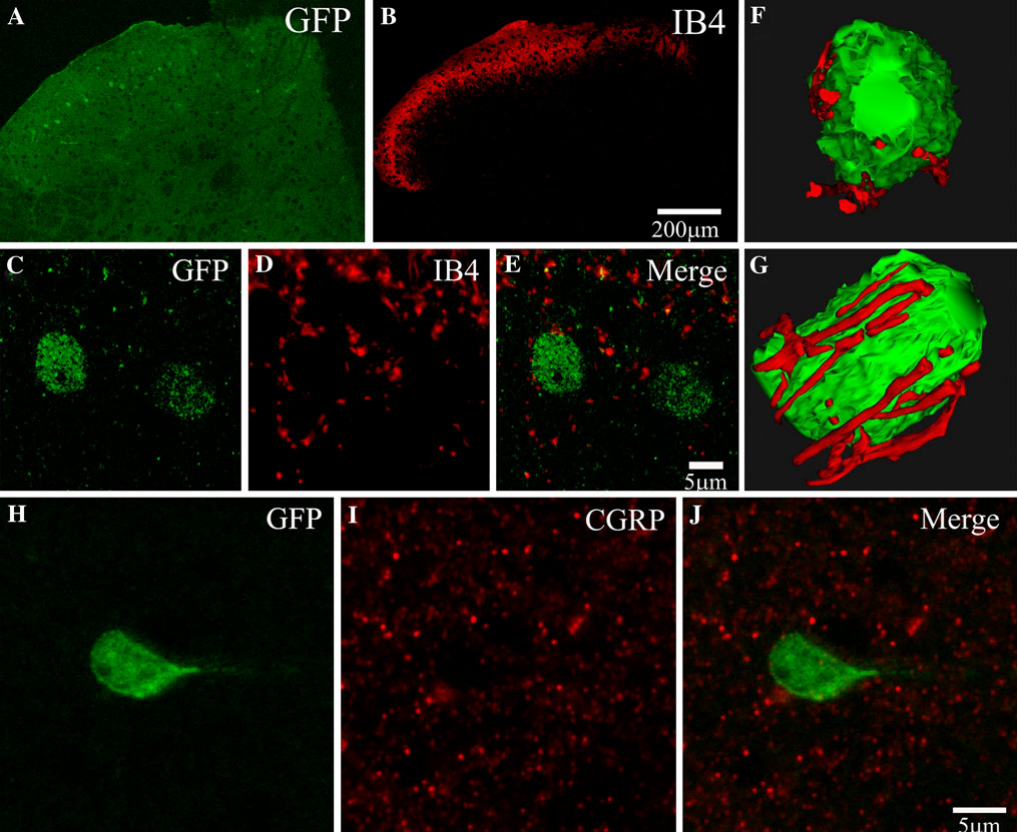
**a**, **b** IB4 staining is clearly observed within laminae I/II of the DH and makes close appositions to both Cx45-eGFP cells within laminae I/II and those in lamina III. **c**–**e** Confocal scan of representative cells (from lamina III in **a**) demonstrate the density of projections surrounding the Cx45-eGFP cells. **f**, **g** A reconstructed model example in two orientations illustrating the density of projections onto the cell soma and putative sites of contact. **h**–**j** Staining with CGRP demonstrates that Cx45-eGFP cells do not receive close appositions from CGRP-positive fibres. Colour images are shown online

To determine putative sources of afferent innervation for Cx45-eGFP cells, immunolabelling for both IB4 (a non-peptidergic fibre marker) and CGRP (a peptidergic fibre marker) was performed. Cx45-eGFP cells within laminae I–II (Fig. 4a, c) were surrounded by IB4-positive fibres and those in lamina III received close appositions from IB4-positive fibres (Fig. 4b, d, e). 3D reconstructed models permitted measurements of putative contact density of IB4-positive fibres onto Cx45-eGFP cell somata (Fig. 4f, g). From 45 Cx45-eGFP cells analysed, the average putative contact density was calculated as 23.6 ± 2.4 appositions per 100 μm^2^. Immunolabelling for CGRP revealed widespread projections of presumed peptidergic sensory neurones predominantly within lamina I and the outer layer of lamina II (Fig. 4i). However, in contrast to IB4, Cx45-eGFP cells were devoid of CGRP-positive close appositions (Fig. 4j).

**Fig. 5:**
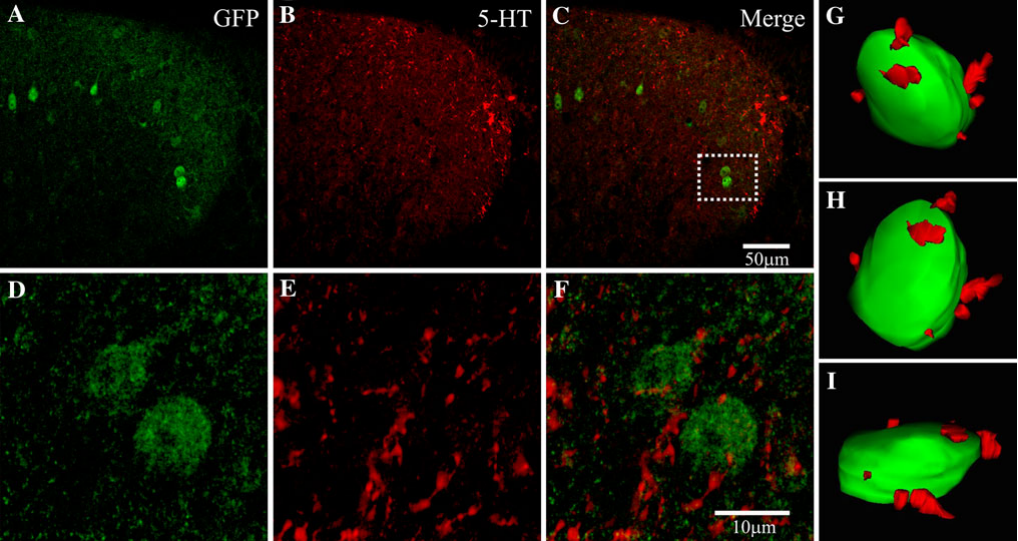
Cx45-eGFP cells receive close appositions from 5-HT positive terminals. Cx45-eGFP can be seen throughout laminae II–III (**a**) and staining for putative 5HT terminals can also be observed throughout the DH (**b**). **d**–**f** High powered magnification of* box A* in **c** displayed two example Cx45-eGFP cells surrounded by a high density of putatively 5-HT terminals. **g**–**i** A reconstructed 3D model example illustrating the density of 5-HT projections surrounding the cell soma and potential sites of contact. Colour images are shown online

Another major extrinsic source of innervation to the dorsal horn is via descending 5-HT pathways. Labelling for this monoamine was widespread throughout the dorsal horn (Fig. 5b, e) and, more specifically, within laminae I–II in the region of Cx45-eGFP cells. 5-HT terminals made close appositions to Cx45-eGFP cell somata (Fig. 5c, f). Using 3D reconstructed models from 45 Cx45-eGFP cells, the contact density of putative 5-HT terminals onto Cx45-eGFP somata was calculated as 15.8 ± 1.92 appositions per 100 μm^2^ (*n* = 3; Fig. 5g–i).

Immunolabelling for either OT or TH confirmed their presence in mouse dorsal horn. Both OT and TH fibres displayed a widespread distribution within the dorsal horn, terminating within laminae I and II and some OT fibres were found in lamina X. However, no clear association with Cx45-eGFP cells was established in this study.

### Cx45-eGFP-IR cells do not contain nNOS, ChAT, NK_1_R, MOR and PKCγ

To characterise the Cx45-eGFP-IR cells further, double immunolabelling was performed with other cell markers for identified dorsal horn populations such as nNOS, ChAT, NK_1_R, MOR and PKCγ (data not shown). Cells immuno-positive for nNOS could be observed throughout the superficial and deep dorsal horn but these were not Cx45-eGFP-IR cells. ChAT-immunoreactivity could be observed within the deep dorsal horn and ventral horn, but did not overlap with Cx45-eGFP-IR. Numerous NK_1_R immunoreactive cells were observed within laminae I and III/IV, but those in lamina III did not co-localize with Cx45-eGFP-IR. The distribution of MOR and PKCγ staining was mainly concentrated within the superficial dorsal horn and also did not overlap with Cx45-eGFP-IR.

## Discussion

In this study, we have demonstrated using Cx45-eGFP transgenic mice that Cx45-eGFP cells are distributed along the rostrocaudal extent of the spinal cord with expression mainly restricted to the grey matter of dorsal laminae I–III. Cx45-eGFP cells were identified as neuronal on the basis of co-localisation with NeuN and confined to a population of interneurones that expressed CB, and to a lesser extent, CR. No association was established between Cx45-eGFP cells and interneurones containing either PV or GAD-67. However, Cx45-eGFP somata were in close apposition to IB4-positive fibres implying a non-peptidergic nociceptive afferent source of peripheral innervation (Snider and McMahon 1998). In addition, Cx45-eGFP cells were in close proximity to 5-HT-containing fibres and may therefore receive some level of monoaminergic descending neuromodulatory input.

### Technical considerations—the Cx45-eGFP reporter mouse model

Localisation of Cx45 protein has been difficult to obtain due to low levels of Cx45 protein and lack of specific antibodies, leading to the study of Cx45 expression using mouse models in which the Cx45 gene has been replaced by that for reporter molecules in the olivo-cerebellar system (Van Der Giessen et al. 2006) and the retina (Maxeiner et al. 2005; Schubert et al. 2005; Dedek et al. 2009). Indeed, cells that express Cx45 as shown by reporter molecules also reveal coupling in retinal neurones typical of gap junctions and this coupling is disrupted when Cx45 is deleted (Maxeiner et al. 2005; Dedek et al. 2009), their Fig. 4e), indicating that reporter expression correlates with Cx45 gap junction protein presence. Such correlation of eGFP expression with Cx45 in the reporter mouse in this study appears to continue in the brain and spinal cord with the observation that mRNA encoding Cx45 and eGFP are present in the thalamic relay neurones and spinal cord ependymal cells (Fig. 1). It cannot be guaranteed that 100 % of cells that express Cx45 can be visualised with eGFP, since it is possible that low levels of expression cannot be visualised even after immunohistochemical amplification. However, similar issues including differentiation from background may also arise with low mRNA levels in in situ hybridisation or low protein levels in immunohistochemistry. Nevertheless, the eGFP reporter mouse facilitates dual immunohistochemical labelling, which is rarely possible under the stringent conditions necessary to detect mRNA using in situ hybridisation. We have therefore utilised this advantage to localise Cx45-eGFP in the spinal cord and to examine the neurochemistry of the eGFP expressing cells in the dorsal horn.

### Cx45 expression in the spinal cord and dorsal horn

Significant numbers of Cx45-eGFP cells were distributed rostrocaudally along the entire spinal cord in cervical, thoracic, lumbar and sacral segments. Cx45-eGFP neurones predominantly lay within superficial dorsal horn, an area crucial for nociceptive sensory processing. The highest concentration of Cx45-eGFP cells was in laminae II–III with fewer cells scattered across lamina I. Cx45-eGFP cells were absent from deeper laminae (IV–VI) which process mainly low threshold non-noxious sensory afferent inputs (Willis and Coggeshall 2004). A strong association of Cx45-eGFP cells in the dorsal horn with the neuronal marker NeuN but not the glial marker GFAP confirms earlier data derived from Cx45-reporter mice, where Cx45 expression was unequivocally associated with neurones rather than glia (Maxeiner et al. 2003). In contrast to previous immunocytochemical data for Cx45 and spinal cord (Pastor et al. 1998), we did not observe significant levels of Cx45-eGFP within white matter oligodendrocytes.

### Co-localisation of Cx45-eGFP cells with CB and CR

Calcium-binding proteins such as CB, CR and PV are abundant in rat spinal dorsal horn and display distinct patterns of expression (Ren and Ruda 1994). In rat superficial dorsal horn, CB-containing cells are distributed across I–III but are especially concentrated within I/II whilst PV-containing cells are concentrated within the inner portion of lamina II as well as laminae III (Yoshida et al. 1990). CR is expressed in neurones of the rat dorsal horn, particularly those localized to laminae II, and more intensely within laminae V and VI (Ren and Ruda 1994). In this study, characterization of CBP expression profiles in mouse dorsal horn revealed close similarities to rat as previously documented (Sasaki et al. 2006), whereby CB and CR expression was observed within laminae I–II, whilst PV intensely stains the inner part of lamina II. A high degree of co-localisation of Cx45-eGFP cells with CB and to a lesser extent with CR but not with PV was evident. The presence of specific CBPs in distinct neuronal populations has prompted the suggestion that CBPs may define specific subsets of interneurones (Antal et al. 1991). Thus, CB is present in glutamatergic neurones whilst PV neurones may characterize a subpopulation of GABAergic interneurones (Ren and Ruda 1994; Todd and Spike 1993; Albuquerque et al. 1999). Based on detected CBP expression profiles, it may be inferred that Cx45-eGFP cells in the mouse dorsal horn are not GABAergic and have an excitatory phenotype. This proposal is reinforced by the lack of co-localisation of Cx45-eGFP cells with GAD-67, a GABA-synthesizing enzyme that is expressed in GABAergic interneurones (Heinke et al. 2004).

In superficial dorsal horn, CB but not PV has been co-localized with neuropeptides such as substance P or enkephalin (Yoshida et al. 1990). Both these neuropeptides operate through G-protein-coupled receptor signalling mechanisms that can impact on intracellular calcium levels and play key roles in nociceptive processing. CB is reportedly present in projection neurones within lamina I that could either be spinothalamic or spinomesencephalic (Yoshida et al. 1990; Menetrey et al. 1992). However, immunolabelling for the NK_1_R receptor, which is present in neurones within lamina I that constitute part of the spinothalamic tract (Al-Khater et al. 2008), revealed no co-localisation with the Cx45-eGFP cells. Nevertheless, some of the CB-containing cells in laminae II–III cannot be ruled out as also projecting to these brain regions (Yoshida et al. 1990).

### Cx45-eGFP cells putatively receive nociceptive non-peptidergic sensory afferent inputs

Two populations of nociceptors that terminate within superficial dorsal horn have been characterized and consist of an IB4-positive non-peptidergic group that projects to the inner layer of lamina II and a CGRP-positive peptidergic group that projects more superficially to lamina I and the outer layer of lamina II (Hunt and Mantyh 2001; Snider and McMahon 1998). There is sparse data on the identity of neurones second order to these groups of sensory afferents, although it is known that peptidergic afferents containing substance P innervate NK_1_R immunoreactive projection neurones in lamina I and those with cell bodies in lamina III or IV (Naim et al. 1997; Todd et al. 2002). In this study, widespread projections of non-peptidergic, presumed nociceptive, IB4-positive afferents were pre-dominantly localized to lamina I and the inner layer of lamina II, with some fibres observed in lamina III. As Cx45-eGFP cells were distributed across laminae I–III, it was observed that all Cx45-eGFP cell bodies within laminae I–II were closely apposed by IB4-positive afferents and most of the Cx45-eGFP cells within lamina III were also in close proximity to IB4-positive afferents. No evidence was obtained for significant putative innervation by the CGRP-positive peptidergic afferent group onto Cx45-eGFP somata although due to the low level of dendritic eGFP expression we cannot eliminate the possibility of undetected dendritic innervation. Thus IB4 nociceptors may provide a significant source of synaptic input to Cx45-eGFP cells in the DH but this requires further validation. Although both groups of nociceptors are physiologically classified as polymodal, i.e. respond to both mechanical and thermal stimuli it has been suggested that IB4 fibres co-expressing the G-protein-coupled receptor ‘Mrgprd’ are selectively required for painful mechanosensation (Cavanaugh et al. 2009; Zylka et al. 2005). In rat dorsal horn, IB4 afferents terminate centrally within glomeruli (Gerke and Plenderleith 2004), typically synapsing within a complex of dendrites and axons surrounded by glia that are believed to provide for pre- and postsynaptic modulation of sensory inputs. Our data for mouse dorsal horn are suggestive of putative contacts between IB4 afferents and Cx45-eGFP neuronal somata rather than within glomeruli but additional electron microscopy will be required to validate this finding and determine whether synapses are present.

Cx45-eGFP cells did not co-localize with PKCγ, a signalling molecule that has been demonstrated to contribute towards the induction and maintenance of persistent pain following injury. PKCγ cells within lamina II in the mouse receive predominantly myelinated afferent inputs (Neumann et al. 2008). As Cx45-eGFP cells do not co-localize PKCγ, this finding complements the proposal that Cx45-eGFP cells receive putative non-peptidergic inputs. Additionally, Cx45-eGFP cells did not co-localize with NK_1_R, MOR, nNOS or ChAT. With the exception of ChAT, all these markers are observed in cells found within either laminae I and/or II and have been documented to be involved with nociceptive processing. ChAT could be observed within lamina III and X, and as cholinergic neurones within lamina III have been shown to frequently co-localize GABA (Spike et al. 1993) this finding ties in with the observed lack of co-localization of GAD-67 with Cx45-eGFP cells.

### Cx45-eGFP cells are innervated by 5-HT-, but not TH- or OT-immunopositive fibres

The superficial dorsal horn receives extrinsic innervation from diverse supraspinal regions via serotoninergic, oxytocinergic, catecholaminergic/dopaminergic descending pathways (VanderHorst and Ulfhake 2006). Immunoreactivity for OT in dorsal horn mainly originates from direct paraventricular hypothalamic projections to the spinal cord (Condes-Lara et al. 2007) and in rat OT-immunoreactivity is predominantly found within laminae I–II (Reiter et al. 1994). TH-immunoreactivity may be representative of noradrenergic, adrenergic and/or dopaminergic fibres that originate in the mesopontine tegmentum especially the locus coeruleus and in the mouse dorsal horn TH-immunoreactivity is most concentrated within laminae II–III (VanderHorst and Ulfhake 2006). In this study, no clear relationship was established between Cx45-eGFP cells and OT-IR or TH-IR terminals. On the other hand, close appositions were detected between 5-HT terminals and Cx45-eGFP cells in laminae I–II. Serotoninergic innervation within the spinal cord is widespread and many 5-HT-IR profiles are found within the inner layer of lamina II with fewer in laminae III/IV (Steinbusch 1981; Marlier et al. 1991). These data are suggestive of innervation of Cx45-eGFP cells via descending monoaminergic control systems that have been described as either pro- or anti-nociceptive depending on the class of 5-HT receptors activated (Suzuki et al. 2004).

### Functional significance of Cx45-eGFP cells in murine dorsal horn nociceptive circuitry

The physiological significance of localization of Cx45 within the superficial dorsal horn or whether Cx45 is assembled into functional gap junctions remains to be resolved. In fact, Cx45 is competent to form heterotypic channels with several other Cx proteins including neuronal Cx36 (Li et al. 2008) and glial Cx43 (Rackauskas et al. 2007). Data from cell lines have demonstrated that Cx45 can form either homotypic (Cx45/Cx45) or heterotypic (Cx45/Cx43) gap junctions that are highly sensitive to the transjunctional voltage (Bukauskas et al. 2002). Previously, using immature rat dorsal horn in vitro, we demonstrated a contribution of gap junctions associated with either neurones or glia in rhythmic behaviour generated by neuronal networks embedded within substantia gelatinosa (Asghar et al. 2005; Chapman et al. 2009). Emerging data reveal a potential contribution of gap junctions to chronic pain states. For example, Cx37 increased in sciatic nerve after injury and this change correlated with behavioural hyperalgesia (Lin et al. 2002). Gap junction-coupled networks may exacerbate neuropathic spreading pain sensations (Spataro et al. 2004) and contribute to abnormal nociceptive behaviours and hyperalgesia induced by inflammation (Qin et al. 2006). In conclusion, it is possible that the dorsal horn expresses Cx protein subtypes that, under certain conditions, are incorporated into functional gap junctions operating within coupled neuronal-glial networks relevant to somatosensory encoding and nociception.

## Abbreviations

5-HT: 5-Hydroxytryptamine
C: Cervical
CC: Central canal
CB: Calbindin
CBP: Calcium-binding proteins
CGRP: Calcitonin gene-related peptide
ChAT: Choline acetyltransferase
CR: Calretinin
Cx: Connexin
DH: Dorsal horn
EAP: Extra-avidin peroxidase
GAD-67: Glutamic acid decarboxylase-67
GFAP: Glial fibrillary-associated protein
eGFP: Enhanced green fluorescent protein
IR: Immunoreactive
ISH: In situ hybridisation
L: Lumbar
NeuN: Neuronal nuclei
NK_1_R: Neurokinin 1 receptor
nNOS: Neuronal nitric oxide synthase
OT: Oxytocin
PBS: Phosphate-buffered saline
PFA: Paraformaldehyde
PGK: Phosphoglycerine kinase
PKCγ: Protein kinase C gamma
PV: Parvalbumin
RTN: Reticular thalamic nucleus
TH: Tyrosine hydroxylase
